# Dopamine D2/3-receptor availability and its association with autonomous motivation to exercise in older adults: An exploratory [^11^C]-raclopride study

**DOI:** 10.3389/fnhum.2022.997131

**Published:** 2022-11-11

**Authors:** Emma Simonsson, Lars Jonasson Stiernman, Anders Lundquist, Erik Rosendahl, Mattias Hedlund, Nina Lindelöf, Carl-Johan Boraxbekk

**Affiliations:** ^1^Department of Community Medicine and Rehabilitation, Physiotherapy, Umeå University, Umeå, Sweden; ^2^Umeå Center for Functional Brain Imaging (UFBI), Umeå University, Umeå, Sweden; ^3^Department of Integrative Medical Biology, Umeå University, Umeå, Sweden; ^4^Department of Statistics, Umeå School of Business, Economics and Statistics, Umeå University, Umeå, Sweden; ^5^Department of Radiation Sciences, Diagnostic Radiology, Umeå University, Umeå, Sweden; ^6^Danish Research Centre for Magnetic Resonance, Centre for Functional and Diagnostic Imaging and Research, Copenhagen University Hospital Amager and Hvidovre, Hvidovre, Denmark; ^7^Department of Neurology, Institute of Sports Medicine Copenhagen (ISMC), Copenhagen University Hospital Bispebjerg, Copenhagen, Denmark; ^8^Faculty of Medical and Health Sciences, Institute for Clinical Medicine, University of Copenhagen, Copenhagen, Denmark

**Keywords:** autonomous motivation, exercise motivation, self-determination theory (SDT), dopamine, PET, aging

## Abstract

**Background:**

Autonomous motivation to exercise occurs when the activity is voluntary and with a perceived inherent satisfaction from the activity itself. It has been suggested that autonomous motivation is related to striatal dopamine D2/3-receptor (D2/3R) availability within the brain. In this study, we hypothesized that D2/3R availability in three striatal regions (nucleus accumbens, caudate nucleus, and putamen) would be positively associated with self-reported autonomous motivation to exercise. We also examined this relationship with additional exploratory analyses across a set of *a priori* extrastriatal regions of interest (ROI).

**Methods:**

Our sample comprised 49 older adults (28 females) between 64 and 78 years of age. The D2/3R availability was quantified from positron emission tomography using the non-displaceable binding potential of [^11^C]-raclopride ligand. The exercise-related autonomous motivation was assessed with the Swedish version of the Behavioral Regulations in Exercise Questionnaire-2.

**Results:**

No significant associations were observed between self-reported autonomous motivation to exercise and D2/3R availability within the striatum (nucleus accumbens, caudate nucleus, and putamen) using semi-partial correlations controlling for ROI volume on D2/3R availability. For exploratory analyses, positive associations were observed for the superior (*r* = 0.289, *p* = 0.023) and middle frontal gyrus (*r* = 0.330, *p* = 0.011), but not for the inferior frontal gyrus, orbitofrontal cortex, anterior cingulate cortex, or anterior insular cortex.

**Conclusion:**

This study could not confirm the suggested link between striatal D2/3R availability and subjective autonomous motivation to exercise among older adults. The exploratory findings, however, propose that frontal brain regions may be involved in the intrinsic regulation of exercise-related behaviors, though this has to be confirmed by future studies using a more suitable ligand and objective measures of physical activity levels.

## Introduction

The beneficial effects of staying physically active throughout life are well established (Lee et al., 2012; Cunningham et al., 2020; Holmlund et al., 2020). Still, many older adults do not manage to engage in the recommended physical activity levels (Hagströmer et al., 2015; Keadle et al., 2016). A fundamental component in why people succeed or fail to engage in exercise is motivation, more specifically, autonomous motivation (Teixeira et al., 2012). According to the Self-Determination Theory (SDT), one of the major motivational frameworks within physical activity and behavioral change (Rhodes et al., 2019), autonomous motivation occurs when activities are engaged for the inherent interest and satisfaction of the activity itself, being voluntary as well as aligned with our personal values (Deci and Ryan, 2000; Ryan and Deci, 2000b). The perceived internal locus of causality is central within SDT and autonomous motivation. While external motivators also facilitate behaviors, such as a monetary reward or avoidance of negative feelings such as shame or guilt, internal motivators generate the more high-quality form of autonomous motivation (Ryan and Deci, 2000b). Sensations such as delight and having fun during the activity are important characteristics of activities taxing autonomous motivation, as well as common reasons for exercising among those older adults that exercise regularly (Dacey et al., 2008; Cancela et al., 2021). Further, autonomous motivation was more positively associated with higher exercise volumes (Teixeira et al., 2012) and long-term adoption of physical exercise among older adults (Kekäläinen et al., 2018).

It has been suggested that the facilitation of autonomous motivation is supported by the dopaminergic system within the brain (Di Domenico and Ryan, 2017). Central to the neurobiology of motivation is the neurotransmitter dopamine (Botvinick and Braver, 2015), and due to its richness in dopamine receptors, the striatum is the brain region most often associated with motivation and reward processing (Haber and Knutson, 2010; Tziortzi et al., 2014). Neuroimaging studies have linked striatal dopamine to internal rewards and traits closely related to autonomous motivation, such as positive affect (Ashby et al., 1999), behavioral perseverance (Salamone and Correa, 2012, 2016), and flow (de Manzano et al., 2013), leading to the proposal that an individual’s capacity for autonomous motivation is related to dopamine receptor availability within the striatum (Di Domenico and Ryan, 2017).

Regarding dopamine and exercise, behavior selection of physical efforts in rodents has been linked to tonic dopamine signaling in the ventral striatum (Niv et al., 2007). Injections of a dopamine antagonist decreased the willingness to perform physical but not cognitive effort among mice (Hosking et al., 2015), and a reduced dopamine D2/3-receptor (D2/3R) availability down-regulated both energy expenditure and physically effortful behaviors among obese mice (Mourra et al., 2020). Similar results were recently detected when modulating dopamine homeostasis in humans (Michely et al., 2020). Also, among older adults, striatal D2/3R availability positively correlates with self-reported physical activity ratings (Köhncke et al., 2018) and cardiorespiratory fitness (Jonasson et al., 2019).

Neuroimaging research on intrinsic regulation of motivation has mainly focused on the striatum, however, there is support also for the involvement of extrastriatal regions such as the frontal cortex, anterior insular cortex, and anterior cingulate cortex (Di Domenico and Ryan, 2017; Cromwell et al., 2020). Even though not examining the direct link to dopamine, functional Magnetic Resonance Imaging (fMRI) studies have observed increased response in these frontal regions during intrinsically motivating settings (Murayama et al., 2010, 2015; Ellwood et al., 2017; Lee and Reeve, 2020). The action of dopamine within these regions has been suggested to be specifically involved in the valuation and consolidation of reward outcome projections from the striatum (Bromberg-Martin et al., 2010; Oldham et al., 2018), processes important for the maintenance and long-term perseverance of motivation (Ryan and Di Domenico, 2016; Myers et al., 2016).

Accordingly, this study aimed to examine the suggested link between dopamine receptor availability and autonomous motivation (Di Domenico and Ryan, 2017). We hypothesized that striatal D2/3R availability would be positively associated with a self-report measurement of exercise-related autonomous motivation among older adults. A secondary aim was to explore this association in *a priori* specified extrastriatal regions (superior frontal gyrus, middle frontal gyrus, inferior frontal gyrus, orbitofrontal gyrus, anterior insular cortex, and the anterior cingulate cortex).

## Materials and methods

### Participants and procedure

The present study used baseline data from the Physical Influences on Brain in Aging (PHIBRA) study (Jonasson et al., 2017). In short, the PHIBRA study recruited sixty older adults (64–78 years) *via* a local newspaper advertisement to participate in a 6-month exercise intervention (aerobic training or stretching and toning control). Exclusion criteria in PHIBRA included having a neurological disease, dopamine-influencing medication, a Mini-Mental State Examination (MMSE) score below 27, diabetes, regularly performing moderately high to high-intensity training, or magnetic resonance imaging (MRI)/positron emission tomography (PET)-incompatible factors such as claustrophobia or metal implants. The Swedish Ethics Review Authority approved the PHIBRA study (Umeå, Sweden; registration number: 2013-238-31M). The study was carried out per the WMA Declaration of Helsinki, and all participants provided written consent before testing.

All participants underwent a baseline data collection distributed on a total of six separate days, described in Jonasson et al. (2017), including the Behavioral Regulations in Exercise Questionnaire-2 (BREQ-2)(Markland and Tobin, 2004) to assess exercise-related motivation, and MRI and PET-scans to assess dopamine D2/3-receptor (D2/3R) availability. By routine, all structural MRI images were screened for abnormalities by a radiologist.

### Autonomous motivation

Exercise-related autonomous motivation was assessed with BREQ-2 (Markland and Tobin, 2004). This questionnaire stems from the organismic integration theory within the SDT, a sub-theory focused on behavior regulation in relation to the quality of motivation (i.e., to what extent motivation is autonomous or controlled) (Ryan and Deci, 2000a). The BREQ-2 consists of 19 items measured on a five-point Likert scale, ranging from 0 (not true for me) to 4 (very true for me), examining five behavior regulation styles (intrinsic, identified, introjected, external and amotivated). The BREQ-2 has been validated with high reliability, and the Swedish version of BREQ-2 has demonstrated Cronbach’s alpha between 0.73 and 0.86 within groups of Swedish adults between 18 and 78 years of age (Weman-Josefsson et al., 2015; Lindwall et al., 2017).

Participants missing no more than one value within a domain were dealt with by imputing the participant’s average score of the items loading on the same motivational construct. Item-aggregation was used to compute a unit-weighted composite score for each motivational construct (Wilson et al., 2012). The scores for the intrinsic and identified constructs were then further averaged into a composite score of autonomous motivation (Standage et al., 2008).

### Neuroimaging

#### Magnetic resonance imaging

Structural MRI data was acquired on a 3T 750 MR scanner (General Electric, WI, US) equipped with a 32-channel head coil. T1-weighted images were acquired using a 3D fast spoiled gradient-echo sequence (180 slices with 1 mm thickness, repetition time (TR) 8.2 ms, time to echo (TE) 3.2 ms, flip angle 12°, field of view 25 × 25 cm).

#### Positron emission tomography

PET data was acquired on a Discovery PET/CT 690 (General Electric, WI, US) using the non-displacable binding potential (BP_*ND*_) of [^11^C]-raclopride ligand to quantify D2/3R availability. Head movements were minimized by individually fitted thermoplastic masks (Positocasts Thermoplastic; CIVCO medical solutions, IA, US). Initially, a low-dose helical CT-scan (20 mA, 120 kV, 0.8 s/revolution) was performed for attenuation-correction purposes. This was followed by an intravenous bolus injection of 250 MBq [^11^C]-raclopride, and a 55-min, 18-frame dynamic PET scan was acquired (9 × 120 s, 3 × 180 s, 3 × 260 s, 3 × 300 s) during resting-state conditions. Attenuation-, scatter- and decay-corrected PET images (slices = 47, field of view = 25 cm, 256 × 256 matrix, voxel size = 0.98 × 0.98 × 3.27 mm3) were reconstructed with the SharpIR algorithm (Bettinardi et al., 2011), and yielded a full width half maximum (FWHM) of 3.2 mm (Wallstén et al., 2013; for more detailed information about the PET-imaging acquisition, see Jonasson et al., 2019; Karalija et al., 2020).

#### Preprocessing

**FIGURE 1:**
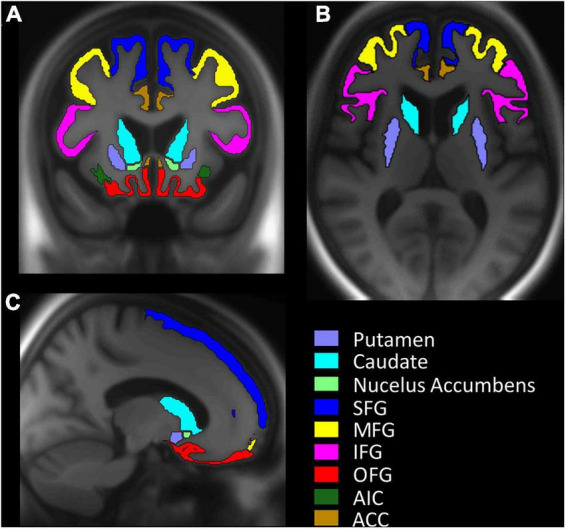
Visualization of gray matter segmentation for each region of interest (ROI). **(A)** Coronal plane. **(B)** Horizontal plane. **(C)** Sagittal plane. SFG, Superior frontal gyrus; MGF, middle frontal gyrus; IFG, inferior frontal gyrus; OFG, orbitofrontal gyrus; AIC, anterior insular cortex; ACC, anterior cingulate cortex.

Processing of T1 images and extraction of cortical (Desikan et al., 2006) and subcortical (Fischl et al., 2002; Destrieux et al., 2010) gray matter segmentation for each region of interest (ROI) was done with Freesurfer version 6 (Figure 1). The *a priori* specified ROIs included the striatum, divided into three sub-regions (nucleus accumbens, caudate nucleus, and putamen). The included extrastriatal regions were based on previous findings of good test-retest reliability for the [^11^C]-raclopride ligand within the PHIBRA Study control group (Karalija et al., 2020), and their suggested involvement in motivational processes (Bromberg-Martin et al., 2010; Di Domenico and Ryan, 2017): superior frontal gyrus (SFG), middle frontal gyrus (MFG; rostral and caudal middle frontal), inferior frontal gyrus (IFG; pars opercularis, pars triangularis, pars orbitalis) and orbitofrontal cortex (OFC; lateral and medial orbitofrontal), the anterior insular cortex (AIC), and the anterior cingulate cortex (ACC; rostral and caudal anterior cingulate).

Motion correction and co-registration of PET images onto T1-weighted images were performed in SPM8. BP_*ND*_ was calculated for each ROI using orthogonal reference Logan analysis (Logan et al., 1996) with linear regression based on the median of ROI voxel values across both hemispheres from each time frame based on frame 10–18 from time 18 to 55 min, with the cerebellum as reference region. See Supplementary Figure 2 for map of [^11^C]-raclopride distribution volume ratio across gray matter.

### Statistical analyses

Data were prepared and analyzed using R version 4.0.5 (R Core Team, 2020) and RStudio version 1.1.463 (RStudio Team, 2016). Plots were produced using the tidyverse (v. 1.3.1) and GridExtra (v. 2.3) packages (Auguie, 2017; Wickham et al., 2019), and semi-partial correlations was performed with the MASS (v. 7.3.53.1) and ppcor (v. 1.1) packages (Kim, 2015).

To examine the association between D2/3R availability and self-reported autonomous motivation, we performed semi-partial correlations on the autonomous motivation composite score and the [^11^C]-raclopride BP_*ND*_ for each of the *a priori* specified regions of interest, controlling for ROI volume on BP_*ND*_ to account for partial volume effects. We assumed a positive direction of the association between D2/3R availability and autonomous motivation and therefore used one-tailed significance testing with an α < 0.05. To control for multiple comparisons among the exploratory analyses, we used the false discovery rate (FDR) (Benjamini and Hochberg, 1995).

## Results

In the present study, we excluded 11 participants due to missing or major incomplete BREQ-2 data. Thus, complete baseline data was obtained from 49 (females, *n* = 28) physically inactive but otherwise healthy older adults (see Table 1). Table 2 presents descriptive data on ROI BP_*ND*_ and Autonomous motivation, with the ROI segmentation visualized in Figure 1.

**TABLE 1:** Sample characteristics.

	Mean (±*SD*)	Min; max
Age (years)	68.7 (2.7)	63.9; 77.7
Years of education	13.8 (4.2)	7.0; 25.0
BMI (kg/m^2^)	26.5 (3.5)	19.4; 37.3
Cardiorespiratory fitness (VO_2_ peak, O_2_ ml/kg × min)	20.4 (3.7)	13.5; 30.0

BMI, Body Mass Index; VO2 peak, peak aerobic capacity assessed during a graded cycle ergometer test, described in detail elsewhere (Jonasson et al., 2017).

**TABLE 2:** Descriptive information of D2/3R availability as [^11^C]-raclopride BP_ND_ for each brain region of interest, and self-reported autonomous motivation.

	Mean ± *SD*	Min; max
**[^11^C]-raclopride BP_ND_ in ROIs**		
**Striatum**		
Nucleus accumbens	2.15 (0.34)	1.40; 2.89
Caudate nucleus	2.22 (0.35)	1.39; 3.00
Putamen	3.39 (0.34)	2.70; 4.01
**Extrastriatal ROIs**		
Superior frontal gyrus (SFG)	0.19 (0.05)	0.10; 0.33
Middle frontal gyrus (MFG)	0.24 (0.05)	0.13; 0.34
Inferior frontal gyrus (IFG)	0.22 (0.05)	0.08; 0.32
Orbitofrontal cortex (OFC)	0.25 (0.05)	0.13; 0.37
Anterior insular cortex (AIC)	0.30 (0.06)	0.15; 0.44
Anterior cingulate cortex (ACC)	0.26 (0.05)	0.13; 0.37
**Exercise motivation**		
Autonomous motivation	2.21 (0.91)	0; 3.75

BP_ND_, non-displaceable binding potential; SD, Standard deviation; Autonomous motivation is a mean composite score based on mean scores for intrinsic and identified motivation.

No significant associations were observed for the semi-partial correlations between D2/3R availability (BP_*ND*_) and self-reported exercise-related autonomous motivation within any of the three striatal regions; nucleus accumbens [*r*(46) = −0.021, *p* = 0.56], caudate [*r*(46) = 0.037, *p* = 0.40], and putamen [*r*(46) = 0.023, *p* = 0.44].

**FIGURE 2:**
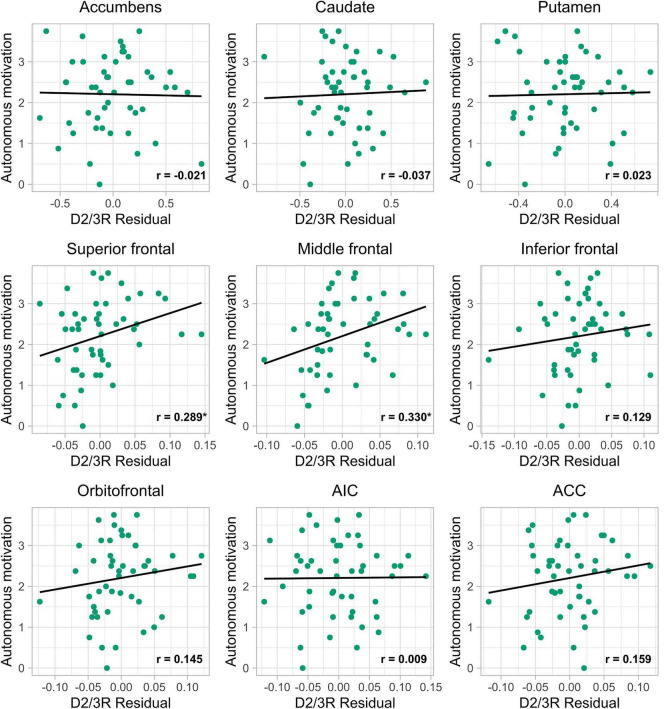
Scatterplots of D2/3R availability within each ROI and autonomous motivation. Self-reported autonomous motivation along the *y*-axis, and the residual (controlling for structural ROI volume) dopamine D2/3-receptor availability (D2/3R residual) estimated with [^11^C]-raclopride positron emission tomography (PET) along the *x*-axis. Nucleus accumbens (accumbens), caudate nucleus (caudate), superior frontal gyrus (superior frontal), middle frontal gyrus (middle frontal), inferior frontal gyrus (inferior frontal), orbitofrontal gyrus (orbitofrontal), anterior insular cortex (AIC), anterior cingulate cortex (ACC). *If *p* < 0.05.

Exploratory analyses for extrastriatal regions revealed a significant positive correlation with the superior frontal gyrus [*r*(46) = 0.289, *p* = 0.023], and the middle frontal gyrus [*r*(46) = 0.330, *p* = 0.011], but not for the inferior frontal gyrus orbitofrontal gyrus, anterior insular cortex, and anterior cingulate cortex. Both significant associations survived an FDR correction of *q* < 0.1. See Table 3 for exact values and statistics, and Figure 2 for scatterplots of autonomous motivation and ROI BP_ND_ residuals (adjusted for ROI volume).

**TABLE 3:** Semi-partial correlations of ROI D2/3R availability measured as [^11^C]-raclopride BP_ND_ and self-reported autonomous motivation.

	Autonomous motivation	
	
	*r*	*p*
**Striatum**		
Nucleus accumbens	–0.021	0.557
Caudate	0.037	0.400
Putamen	0.023	0.438
**Extrastriatal ROIs**		
Superior frontal gyrus (SFG)	0.289	0.023*
Middle frontal gyrus (MFG)	0.330	0.011*
Inferior frontal gyrus (IFG)	0.129	0.190
Orbitofrontal cortex (OFC)	0.145	0.162
Anterior insular cortex (AIC)	0.010	0.474
Anterior cingulate cortex (ACC)	0.159	0.140

BP_ND_, non-displaceable binding potential; *p* < 0.05 in bold face.

*If significant also after FDR correction (*q* < 0.1).

Results remained essentially the same for *post hoc* sensitivity analyses not adjusting for ROI-volume (see Supplementary Table 1 for test statistics, and Supplementary Figure 1 for scatterplots of autonomous motivation and raw BP_ND_ data), adjusting for ROI-volume, age, and sex (Supplementary Table 2), or removing two outliers (≥ 2.5 *SD* in BP_ND_ change) detected during examination of longitudinal data/repeated measurements (Supplementary Table 3 controlling for ROI-volume, and Supplementary Table 4 controlling for ROI-volume, age, and sex).

An additional correlation between participants’ cardiorespiratory fitness level (VO_2_ peak) and self-reported autonomous motivation to exercise did not reach statistical significance [*r*(47) = 0.194, *p* = 0.181], neither when adjusted for age and sex [*r*(45) = 0.188, *p* = 0.205].

## Discussion

This study examined the relationship between dopamine and autonomous motivation to exercise among older adults. We specifically aimed to examine the suggested link between D2/3R availability in the striatum and exercise-related autonomous motivation. We found no support for the hypothesis that D2/3R availability was to be positively associated with self-reported exercise-related autonomous motivation in neither of the three striatal regions (nucleus accumbens, caudate nucleus, and putamen). Exploratory analyses revealed statistically significant positive associations for the superior frontal gyrus and the medial frontal gyrus, but not for the inferior frontal gyrus, orbitofrontal gyrus, anterior insular cortex, or anterior cingulate cortex.

The absence of significant associations between striatal D2/3R availability and self-reported autonomous motivation to exercise may be explained by the striatum being more involved in acute reward processes during an activity (Bromberg-Martin et al., 2010; Olivetti et al., 2019). Striatal dopamine acts upon intrinsic and extrinsic reward stimuli (Jonasson et al., 2014; Oldham et al., 2018), and assessment of resting dopamine D2/3R availability in the striatum might not be enough to disentangle the specific influence on autonomous regulation. Also, while the acute processes are important in the generation phase of motivation, influencing maintenance and regulation of motivation, these acute aspects might not be covered by the BREQ-2 questionnaire (Markland and Tobin, 2004). This questionnaire focuses on why people engage in exercise, possibly only capturing already existing mental representations of previous exercise-related experiences necessary for future engagement of similar behaviors (Lee and Reeve, 2020). To explore the role of striatal D2/3R availability during the acute phase of internal reward processes and autonomous motivation, future studies PET studies should use a different study design, possibly including a memory recall or imaginary task designed to target autonomous motivation (Lee and Reeve, 2020). In addition, other reward-governing neurotransmitter systems influenced by exercise, for example involving opioids (Saanijoki et al., 2018) or endocannabinoids (Siebers et al., 2021) may also be relevant to explore in relation to autonomous exercise-related motivation.

Our results from the exploratory analyses suggest that D2/3R availability in the superior and middle frontal gyrus may be involved in the intrinsic regulation of autonomous motivation to exercise. These frontal regions may support the maintenance and long-term perseverance of autonomous motivation *via* dopamine-dependent salience processes of attention orientation toward consolidated positive memories relevant for decision-making to perform a similar activity again (Bromberg-Martin et al., 2010; Ott and Nieder, 2019). A possible explanation for why autonomously motivated individuals more successfully maintain a physically active lifestyle over time (Ryan and Deci, 2000b; Teixeira et al., 2012; Kekäläinen et al., 2018).

The suggested relationship between dopamine and autonomous motivation to exercise becomes particularly interesting in light of the known age-related deteriorations of the dopaminergic system (Karrer et al., 2017; Karalija et al., 2019). Notably, older adults with higher physical activity levels show less than expected age-related reduction in D2/3R availability (Dang et al., 2017), and inter-individual variation in dopamine-related genes that affects dopamine receptor efficacy was associated with less time spent in moderate to vigorous physical activity, and even more so among the oldest (Rosso et al., 2018; Dohrn et al., 2020). Thus, while physical exercise may have the potential to maintain or even strengthen the integrity of the dopamine system, aging as well as a person’s genetic makeup may cause difficulties in engaging in such activities due to decreased D2/3R efficacy.

The present study design cannot determine causality, and we do not address the potential exercise-related changes that might occur over time, such as plasticity mechanisms within the dopaminergic system (Jonasson et al., 2019), or the motivational profile (Kekäläinen et al., 2018). Still, we propose that individuals with higher D2/3R availability in frontal regions can, to a greater extent, rely more on autonomous motivation processes. In contrast, those with lower levels might be more dependent on controlled processes and external motivators. In addition to other factors important for successful exercise routines [e.g., environmental settings, external expectations, consolidated habits or social norms (Pelssers et al., 2018; Aaltonen et al., 2020; Jones et al., 2020; Cancela et al., 2021)], future studies should acknowledge that the individual variation in motivation might also be linked to biological differences in the dopaminergic system, and that aging *per se* might affect this system and shift the motivational profile (Yee et al., 2019).

It should be noted that the absence of associations between striatal D2/3R and subjective exercise-related autonomous motivation, and between autonomous motivation and VO_2_ peak, were despite a significant relationship between striatal D2/3R and VO_2_ peak previously observed (Jonasson et al., 2019). Striatal D2/3R among older adults has also been related to self-reported physical activity, with a positive association for higher levels of physical activity intensity but not physical activity volume (Köhncke et al., 2018). Possibly, frontal D2/3R and autonomous motivation operate in a parallel process to that of striatal D2/3R and physical fitness, with frontal D2/3R more involved in whether exercise will be performed or not (i.e., volume) rather than how it is performed (i.e., intensity). These remaining questions need to be addressed in future studies, in which it also should be included additional objective measures of physical activity, such as accelerometer data, to examine the intricate interplay of dopamine, motivation to exercise, and different activity levels.

### Limitations

This study has limitations. First, the validity of [^11^C]-raclopride for BP_ND_ quantification in extrastriatal regions has recently been questioned (Svensson et al., 2019; Freiburghaus et al., 2021), emphasizing that the interpretation of [^11^C]-raclopride BP_ND_ in frontal regions should be made with caution (Freiburghaus et al., 2021). There is, however, evidence of high test-retest reliability (Alakurtti et al., 2015; Karalija et al., 2020), good measurement properties when linking functionally connected cortical areas in latent space (Papenberg et al., 2019), and strong linear relationships between [^11^C]-raclopride D2/3R estimates in cortical regions (Papenberg et al., 2019) with D2/3R measured in the post-mortem brain (Hall et al., 1994), all which speaks to the feasibility of using [^11^C]-raclopride to assess extrastriatal D2/3R in the living brain. Of note, in a subsample of the current study sample, the extrastriatal regions included have presented high test-retest reliability (superior frontal gurys, ICC: 0.93, *r* = 0.88; middle frontal gurys, ICC: 0.96, *r* = 0.93), and a coefficient of variance between 18.4 and 24.5% (Karalija et al., 2020). Still, we recommend that future studies examining extrastriatal D2/3R include a ligand with higher sensitivity, such as [^11^C]FLB457 (Freiburghaus et al., 2021). Second, with the [^11^C]-raclopride BP_ND_ we only assess receptor availability and therefore cannot conclude about the number of receptors (i.e., density), nor about the level of endogenous dopamine bound to the receptors. Hence, if motivated subjects have higher resting synaptic dopamine levels, this could potentially reduce D2/3R availability in these individuals and consequently reduce the estimated association between D2/3R availability and autonomous motivation. Third, our sample consisted of a group of non-exercising older adults that were interested (and motivated) in participating in a 6-month exercise intervention. Although our sample portrayed the whole range of autonomous motivation with BREQ-2 scores ranging from low to high, all participants had some degree of motivation to participate in an exercise intervention and this may have narrowed the autonomous motivation range in this study. Further exploration of autonomous motivation in relation to dopamine should also be done within more heterogeneous groups of older individuals. Fourth, a larger sample size would allow for the possibility of performing subgroup analyses not possible in the present study, such as comparing the different domains within the SDT (Chemolli and Gagné, 2014), genetic variations, gender differences, and physical activity levels (Dacey et al., 2008; Aaltonen et al., 2020). Finally, although widely used, the BREQ-2 questionnaire (Markland and Tobin, 2004) excludes the third regulation style of autonomous motivation in the SDT, integrated motivation. This part of the autonomous motivation spectrum has been considered highly relevant for maintaining physical activity and exercise (Wilson et al., 2012; Miquelon and Castonguay, 2017). However, concerns regarding high inter-factor correlations favor assessment only of the intrinsic and identified scales, as is done in the BREQ-2 questionnaire (Howard et al., 2017). In summary, future studies could preferably include additional measures of dopamine, larger sample sizes, and alternative designs to further explore and disentangle the biological mechanisms of autonomous motivation.

## Conclusion

To summarize, D2/3R availability in the striatum was not associated with self-reported exercise-related autonomous motivation. The exploratory analyses suggest that D2/3R availability in the superior and middle frontal gyrus may be involved in the intrinsic regulation of exercise-related behaviors, potentially *via* salient processes enhancing decision-making and long-term maintenance. This suggestion should preferably be confirmed in larger studies, including a different assessment of D2/3R availability. Still, studies aimed at increasing motivation and engagement in physical activity among older adults could benefit from considering the possible variations in the support needed due to aging and inter-individual biological differences in dopamine. While some individuals might have better prerequisites to develop and consolidate autonomous forms of motivation to exercise, others might need more external support over time.

## Data availability statement

The datasets used and analyzed during the current study are available from the corresponding author on reasonable request. Requests to access these datasets should be directed to ES, emma.simonsson@umu.se.

## Ethics statement

The studies involving human participants were reviewed and approved by the Swedish Ethics Review Authority (Umeå, Sweden; registration number: 2013-238-111 31M). The patients/participants provided their written informed consent to participate in this study.

## Author contributions

ES performed the statistical analyses, initial interpretations of the results, and drafting of the manuscript. C-JB contributed during analyses, interpretations of the results, and editing of the manuscript. AL assisted during the analyses and interpretations of the results. LJS, ER, MH, and NL contributed equally to the interpretations of the results and editing of the manuscript. All authors read and approved the final manuscript.
