# Supplementary material for: Dopamine D2/3-receptor availability and its association with autonomous motivation to exercise in older adults: An exploratory [11C]-raclopride study
Source: Front Hum Neurosci. 2022 Nov 11;16:997131. doi: 10.3389/fnhum.2022.997131 (PMC9691986; doi:10.3389/fnhum.2022.997131)

Supplementary Material

# Supplementary Data

# Analysis without adjustment of ROI volume

## Supplementary Table 1

Analyses without correcting for ROI volume. Results essentially the same.

| **Supplementary Table 1.** Correlations of D2/3R availability measured as ^11^C-raclopride BP_ND_ and self-reported autonomous motivation, without controlling for ROI volume | | |
| --- | --- | --- |
| **Regions of Interest** | **Autonomous motivation** | |
| **Striatum** | r | p |
| Nucleus accumbens | -0.007 | 0.519 |
| Caudate | 0.02 | 0.446 |
| Putamen | -0.02 | 0.554 |
| **Extrastriatal ROIs** |  |  |
| Superior frontal gyrus (SFG) | 0.283 | **0.024*** |
| Middle frontal gyrus (MFG) | 0.323 | **0.012*** |
| Inferior frontal gyrus (IFG) | 0.126 | 0.195 |
| Orbitofrontal cortex (OFC) | 0.144 | 0.161 |
| Anterior Insular Cortex (AIC) | 0.017 | 0.453 |
| Anterior Cingulate Cortex (ACC) | 0.16 | 0.135 |
| *Note.* BP_ND_: non-displaceable binding potential, SD: Standard deviation; p < 0.05 in bold face. *significant also after FDR-correction (q < .1). | | |

## Supplementary Figure 1

**Supplementary Figure 1.** Scatterplots of D2/3R availability and autonomous motivation, using raw BP-data instead of BP-residuals controlling for ROI volume.


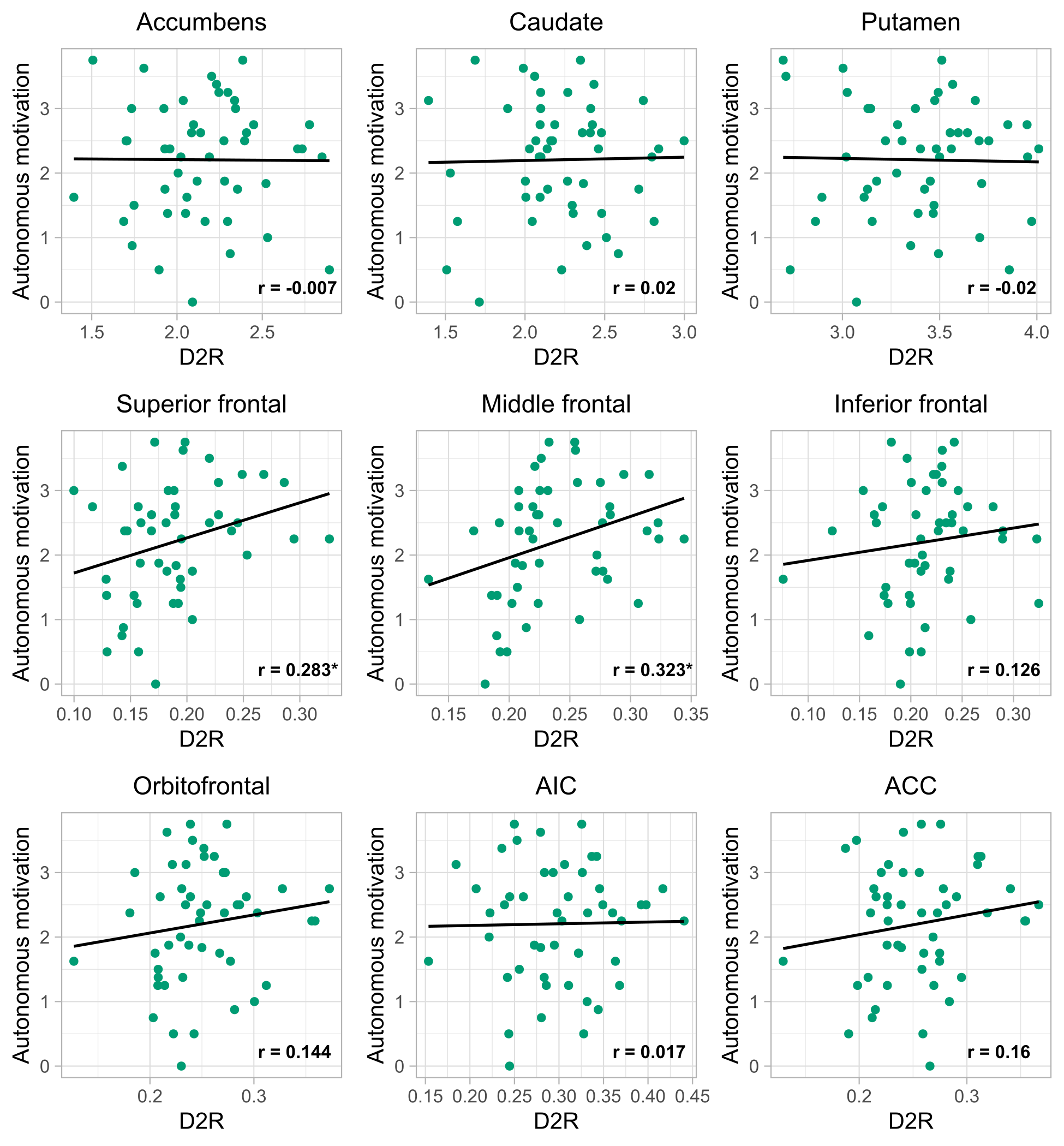


# Analysis with adjustment: Age + Sex

## Supplementary Table 2

Adjusting for ROI-volume, age and sex, results are still essentially the same.

| **Supplementary Table 2.** Semi-partial correlations of D2/3R availability measured as ^11^C-raclopride BP_ND_ and self-reported autonomous motivation adjusted also for age and sex | | |
| --- | --- | --- |
| **Regions of Interest** | **Autonomous motivation** | |
| **Striatum** | r | p |
| Nucleus accumbens | -.025 | .567 |
| Caudate | .014 | .464 |
| Putamen | .022 | .443 |
| **Extrastriatal ROIs** |  |  |
| Superior frontal gyrus (SFG) | .276 | **.032*** |
| Middle frontal gyrus (MFG) | .326 | **.013*** |
| Inferior frontal gyrus (IFG) | .149 | .162 |
| Orbitofrontal cortex (OFC) | .143 | .171 |
| Anterior Insular Cortex (AIC) | -.037 | .596 |
| Anterior Cingulate Cortex (ACC) | .152 | .157 |
| *Note.* BP_ND_: non-displaceable binding potential, SD: Standard deviation; p < 0.05 in bold face. *significant also after FDR-correction (q < .1). | | |

# Analysis with outlier removal

This study focused on baseline data from the PHIBRA-study, however examination of participant BP data from baseline and follow-up revealed two participants with extreme changes/variability across the two time points (≥ 2.5 SD in BP_ND_ change). Although no issues of the co-registration for the two participants were detected, we chose to perform a post hoc sensitivity analysis without these potential outliers. Results still remained essentially the same, though with a stronger association for superior frontal gyrus (see Supplementary Table 3).

## Supplementary Table 3

| **Supplementary Table 3** Semi-partial correlations of D2/3R availability measured as ^11^C-raclopride BP_ND_ and self-reported autonomous motivation without potential outliers | | |
| --- | --- | --- |
| **Regions of Interest** | **Autonomous motivation** | |
| **Striatum** | r | p |
| Nucleus accumbens | 0.012 | 0.467 |
| Caudate | 0.048 | 0.375 |
| Putamen | 0.032 | 0.417 |
| **Extrastriatal ROIs** |  |  |
| Superior frontal gyrus (SFG) | 0.336 | **0.011*** |
| Middle frontal gyrus (MFG) | 0.339 | **0.011*** |
| Inferior frontal gyrus (IFG) | 0.117 | 0.22 |
| Orbitofrontal cortex (OFC) | 0.133 | 0.188 |
| Anterior Insular Cortex (AIC) | -0.013 | 0.534 |
| Anterior Cingulate Cortex (ACC) | 0.186 | 0.108 |
| *Note.* BP_ND_: non-displaceable binding potential, SD: Standard deviation; p < 0.05 in bold face. *significant also after FDR-correction (q < .1). | | |

## Supplementary Table 4

Similarly, results remained essentially the same without these two potential outliers/measurement errors, also when adjusting for age and sex.

| **Supplementary Table 4** Semi-partial correlations of D2/3R availability measured as ^11^C-raclopride BP_ND_ and self-reported autonomous motivation adjusted also for age and sex, and without two potential outliers/measurement errors | | |
| --- | --- | --- |
| **Regions of Interest** | **Autonomous motivation** | |
| **Striatum** | r | p |
| Nucleus accumbens | 0.009 | 0.477 |
| Caudate | 0.026 | 0.433 |
| Putamen | 0.036 | 0.407 |
| **Extrastriatal ROIs** |  |  |
| Superior frontal gyrus (SFG) | 0.334 | **0.013*** |
| Middle frontal gyrus (MFG) | 0.342 | **0.012*** |
| Inferior frontal gyrus (IFG) | 0.146 | 0.173 |
| Orbitofrontal cortex (OFC) | 0.14 | 0.182 |
| Anterior Insular Cortex (AIC) | -0.046 | 0.616 |
| Anterior Cingulate Cortex (ACC) | 0.182 | 0.118 |
| *Note.* BP_ND_: non-displaceable binding potential, SD: Standard deviation; p < 0.05 in bold face. *significant also after FDR-correction (q < .1). | | |

# Supplementary Figure 2: Map of [11C]-raclopride distribution volume ratio across gray matter

**Supplementary Figure 2. Map of [11C]-raclopride distribution volume ratio across gray matter.** The distribution volume ratio (DVR) of [11C]-raclopride was overlaid on a gray matter mask (provided in spm12) with a 0.5 probability threshold. The color scale (red to yellow) denotes the DVR from 1 to 1.5.


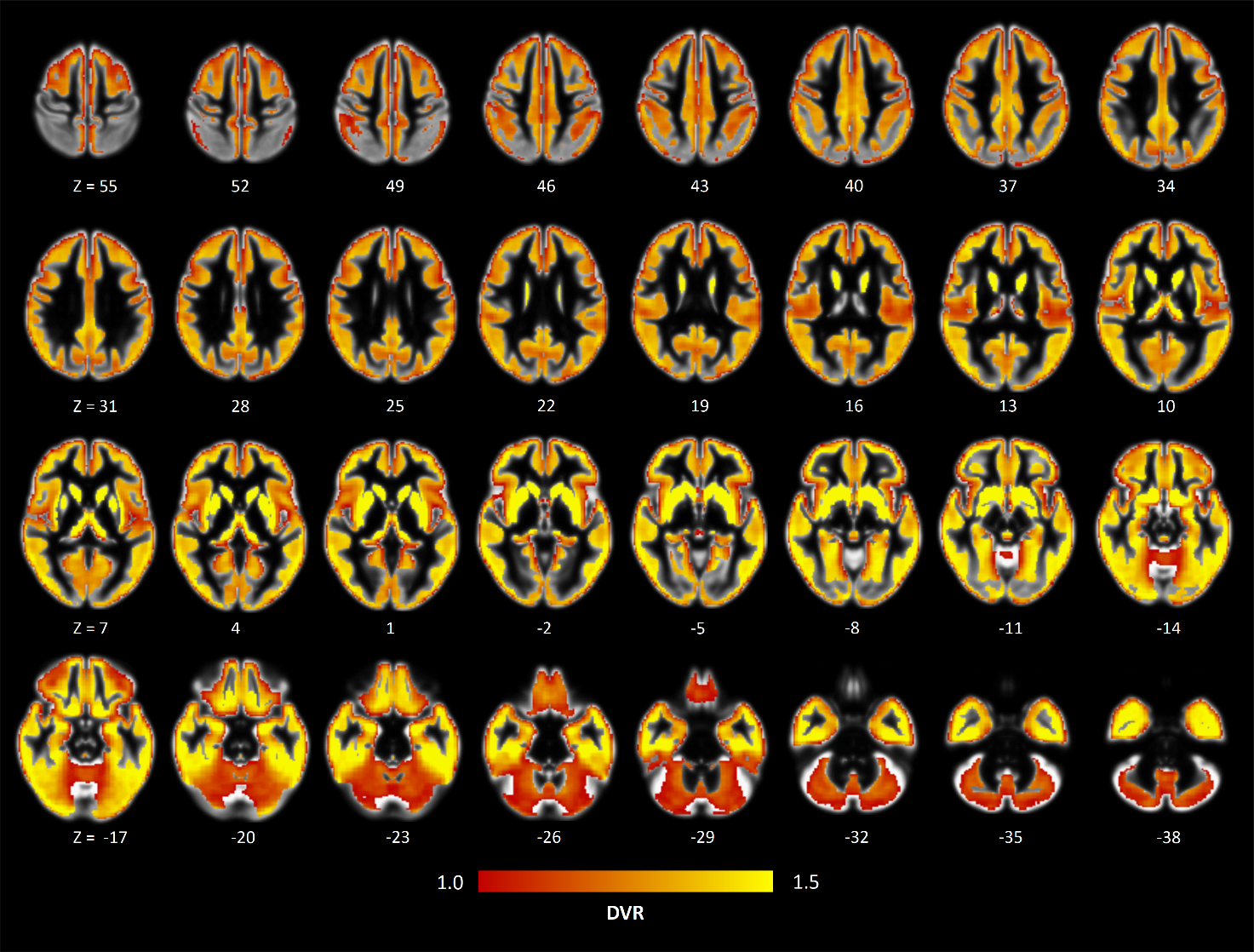

Supplement: Supplementary file 1 [file Data_Sheet_1.docx]
